# Interface Optimization, Microstructural Characterization, and Mechanical Performance of CuCrZr/GH4169 Multi-Material Structures Manufactured via LPBF-LDED Integrated Additive Manufacturing

**DOI:** 10.3390/ma18102206

**Published:** 2025-05-10

**Authors:** Di Wang, Jiale Lv, Zhenyu Liu, Linqing Liu, Yang Wei, Cheng Chang, Wei Zhou, Yingjie Zhang, Changjun Han

**Affiliations:** 1 School of Mechanical and Automotive Engineering, South China University of Technology, Guangzhou 510641, China; mewdlaser@scut.edu.cn (D.W.); 202220100686@mail.scut.edu.cn (J.L.); zliu362-c@my.cityu.edu.hk (Z.L.); melinqingliu@mail.scut.edu.cn (L.L.); weiyang_scuter@163.com (Y.W.); 2 Guangdong-Hong Kong Joint Laboratory of Modern Surface Engineering Technology, Institute of New Materials, Guangdong Academy of Sciences, Guangzhou 510651, China; changcheng@gdinm.com; 3 Guangdong Provincial Key Laboratory of Modern Surface Engineering Technology, Institute of New Materials, Guangdong Academy of Sciences, Guangzhou 510651, China; 4 Department of Mechanical and Electrical Engineering, Xiamen University, Xiamen 361005, China; weizhou@xmu.edu.cn; 5 Shien-Ming Wu School of Intelligent Engineering, South China University of Technology, Guangzhou 511442, China

**Keywords:** multi-material additive manufacturing, LPBF-LDED integrated additive manufacturing, CuCrZr/GH4169, interfacial bonding

## Abstract

CuCrZr/GH4169 multi-material structures combine the high thermal conductivity of copper alloys with the high strength of nickel-based superalloys, making them suitable for aerospace components that require efficient heat dissipation and high strength. However, additive manufacturing of such dissimilar metals faces challenges, with each laser powder bed fusion (LPBF) and laser directed energy deposition (LDED) process having its limitations. This study employed an LPBF-LDED integrated additive manufacturing (LLIAM) approach to fabricate CuCrZr/GH4169 components. CuCrZr segments were first produced by LPBF, followed by LDED deposition of GH4169 layers using optimized laser parameters. The microstructure, composition, and mechanical properties of the fabricated components were analyzed. Results show a sound metallurgical bond at the CuCrZr/GH4169 interface with minimal porosity and cracks (typical defects at the interface), achieved by exceeding a threshold laser energy density. Elemental interdiffusion forms a 100–200 μm transition zone, with a smooth hardness gradient (97 HV0.2 to 240 HV0.2). Optimized specimens exhibit tensile failure in the CuCrZr region (234 MPa), confirming robust interfacial bonding. These findings demonstrate LLIAM’s feasibility for CuCrZr/GH4169 and underscore the importance of balancing thermal conductivity and mechanical strength in multi-material components. These findings provide guidance for manufacturing aerospace components with both high thermal conductivity and high strength.

## 1. Introduction

Multi-material metal structures, achieved by tailoring the spatial arrangement of diverse materials, can simultaneously deliver multifunctional attributes (e.g., magnetism, high thermal conductivity, high-temperature oxidation resistance, and corrosion resistance) along with superior mechanical properties (e.g., a balanced combination of strength and ductility, high hardness, and excellent wear resistance). This synergistic approach offers significant potential for enhancing the stability and service life of metal components operating under extreme conditions [1]. Moreover, multi-material structures that integrate copper-based alloys with nickel-based superalloys combine high thermal conductivity with exceptional strength, making them particularly well-suited for aeroengine components that demand outstanding thermal performance, resistance to thermal fatigue, and high strength [2,3,4].

Additive manufacturing (AM) has emerged as a transformative technology for multi-material fabrication, providing unparalleled design flexibility and rapid prototyping capabilities. In particular, two key AM techniques—laser powder bed fusion (LPBF) and laser directed energy deposition (LDED)—have been extensively investigated for fabricating complex structures [5]. Repnin et al. [6] manufactured a multi-material structure of Inconel 718/Ti6Al4V via LPBF, employing Cu and Nb as interlayers, and achieved an ultimate tensile strength of 910 MPa. Melzer et al. [7] produced 316L/Inconel 718 multi-material specimens through alternating deposition using LDED and subsequently investigated their mechanical and structural properties.

LPBF provides high-resolution details and excellent surface finish, making it an ideal choice for fabricating complex components [8]. However, its effectiveness is often hindered by a limited build volume. In contrast, LDED excels at depositing materials over larger areas at high deposition rates, which is advantageous for fabricating large-scale or functionally graded components. Nonetheless, compared with LPBF, LDED typically offers lower dimensional accuracy and a coarser surface finish [9]. Based on these insights, LPBF-LDED integrated additive manufacturing (LLIAM) is emerging as a promising strategy for fabricating large multi-material components with intricate structural details, meeting the stringent requirements of aerospace applications, especially for hot-section engine parts such as combustors and turbine blades [10,11]. Zhang et al. [12] successfully manufactured a bimetallic structure of QCr0.8 Cu alloy and S06 stainless steel with an In718 interlayer using LLIAM. Bettencourt [13] produced In625 on an LPBF manufactured SS316L matrix via LDED, employing both direct bonding and gradient transition approaches, and subsequently investigated the elemental distribution and related properties in the bonding region. Montero-Sistiaga et al. [14] used LPBF to manufacture the inner part of the check valve (In718), which requires high corrosion resistance, and then used a more economical material (316L) to manufacture the outer part of the check valve through LDED. This technical route significantly shortened the manufacturing time while producing larger, multi-material, and complex components. However, the LLIAM of multi-material components has not been extensively studied, and further research on the compatibility of multi-materials and performance optimization is urgently required.

CuCrZr is a precipitation-hardened copper-based alloy. By adding a small amount of chromium and zirconium to copper (forming a strengthening phase), the alloy achieves a combination of high strength, high thermal conductivity, and good fatigue resistance. GH4169 is a nickel-based precipitation-strengthened superalloy, which is known for its excellent high-temperature strength, good oxidation resistance and fatigue resistance. CuCrZr/GH4169 multi-material components have unique advantages in balancing the high thermal conductivity and high-temperature strength of components, making them very promising for aerospace applications. For example, the regenerative cooling combustion chamber of a liquid rocket engine contains a complex internal liquid-fuel flow channel that must simultaneously meet requirements for efficient heat dissipation and high-temperature strength [15]. Such a chamber is ideally manufactured using CuCrZr/GH4169 multi-material construction, as this material combination provides both high thermal conductivity and high-temperature strength. For the complex-structured CuCrZr inner wall, LPBF is suitable, whereas the outer wall is fabricated via LDED using GH4169 to improve the component’s strength and fabrication efficiency. To date, there have been studies on fabricating CuCrZr/GH4169 multi-materials via LPBF [16], but research on applying LLIAM to CuCrZr/GH4169 multi-materials remains nonexistent. LLIAM of CuCrZr/GH4169 multi-materials faces many challenges. First, the high thermal conductivity and high reflectivity of CuCrZr make it difficult to achieve a metallurgical bond at the interface. Second, the different thermal expansion coefficients of the two materials can easily lead to high thermal stress during cooling. Furthermore, the inhomogeneity of the microstructure and composition has an adverse effect on the mechanical properties [17,18]. Together, these challenges create an urgent need to investigate the compatibility, bonding process, interfacial microstructure, and performance of CuCrZr/GH4169 multi-material combinations in LLIAM.

To address the aforementioned issues, this study investigates the formability of CuCrZr/GH4169 multi-material components using LLIAM. Furthermore, the manufacturing process parameters at the multi-material interface are optimized, and the effects along with the underlying mechanisms of these parameters on the microstructure and properties—particularly in the interfacial bonding region—are systematically examined. This work is intended to serve as a reference for further development and application of LLIAM technology and CuCrZr/GH4169 multi-material components.

## 2. Materials and Methods

### 2.1. Materials and Equipment

**Figure 1:**
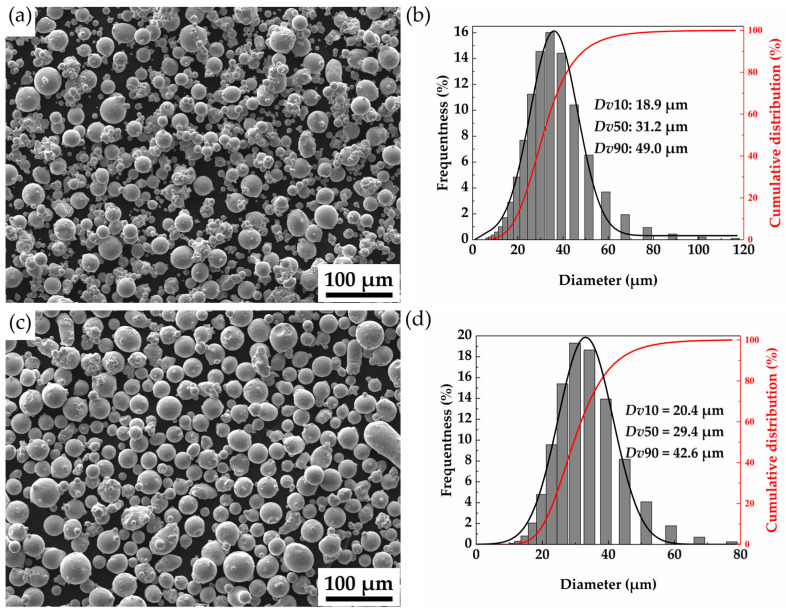
Microstructural morphology and particle size distribution statistics of CuCrZr and GH4169 powders: (**a**,**b**) CuCrZr powder; (**c**,**d**) GH4169 powder.

The metal powder materials used in this study are CuCrZr powder produced by Jiangsu Vilory Advanced Materials Technology Co., Ltd. (Xuzhou, China) and GH4169 powder produced by Avimetal Powder Metallurgy Technology Co., Ltd. (Beijing, China) The specific compositions of the two materials are shown in Table 1. Figure 1 shows the microscopic morphology and particle size distribution of the metal powder used, where Dv10, Dv50 and Dv90 represent the particle diameters when the cumulative volume accounts for 10%, 50% and 90% of the powder particle size distribution, respectively. Both powders have good sphericity.

**Figure 2:**
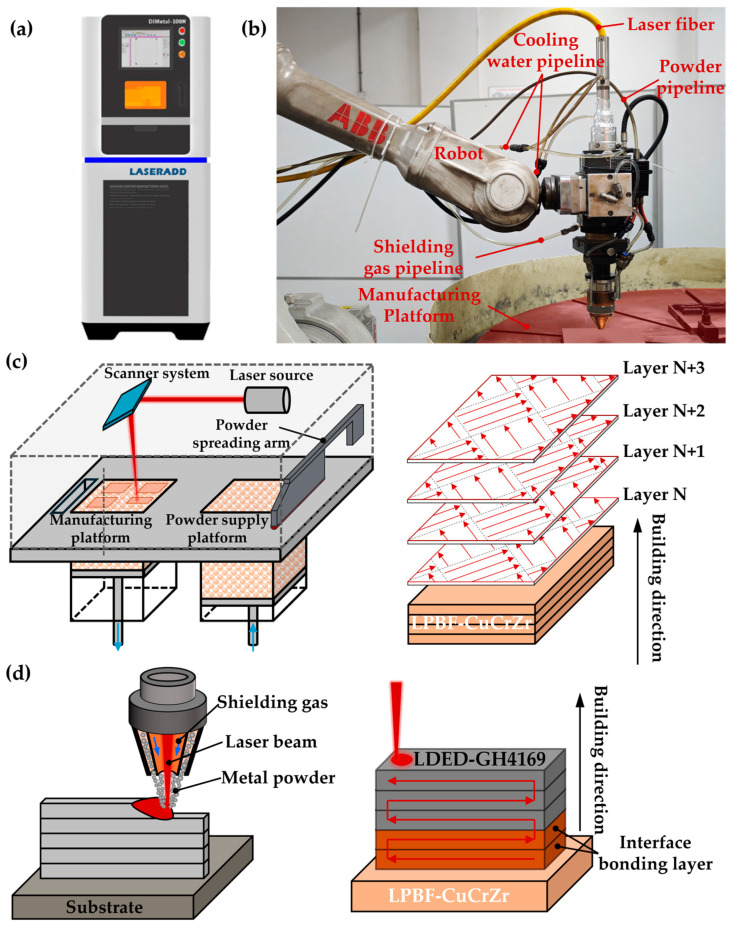
Equipment and principles: (**a**) SLM equipment; (**b**) LDED equipment; (**c**) SLM manufacturing principle and laser scanning strategy; (**d**) LDED deposition principle and laser scanning strategy.

The metal AM equipment utilized in this study includes the DiMetal-100H LPBF device (South China University of Technology, Guangzhou, China) and the robotic LDED AM system (South China University of Technology, Guangzhou, China). The physical representations of these systems are illustrated in Figure 2a,b. The DiMetal-100H is equipped with a 500 W infrared Yb-doped fiber laser, featuring a focused spot diameter of 60 to 80 μm which enables the LPBF of highly reflective materials such as CuCrZr. The robotic LDED AM system primarily comprises a six-axis robot, a laser optical system, a powder feeding system, a water-cooling system, a gas-protection system, and a work platform. It is equipped with a 6000 W semiconductor laser, with a spot diameter of 3 to 4 mm, and the robot drives the laser deposition nozzle to achieve LDED manufacturing.

### 2.2. Experimental Methods

The process principles of the LLIAM of CuCrZr/GH4169 are illustrated in Figure 2c,d. Initially, a bulk CuCrZr component is manufactured using the LPBF, followed by the deposition of GH4169 onto the CuCrZr matrix via LDED. To achieve high-quality specimens, it is essential to optimize the process parameters at the interface of the LLIAM of CuCrZr/GH4169. Because the LPBF process parameters have minimal impact on the interfacial bonding of CuCrZr, this study focuses on the process parameters affecting the interfacial bonding of the two materials during LDED. The parameters selected for the manufacture of single melt track, single layer, and thin-walled solid are as shown in Table 2.

**Figure 3:**
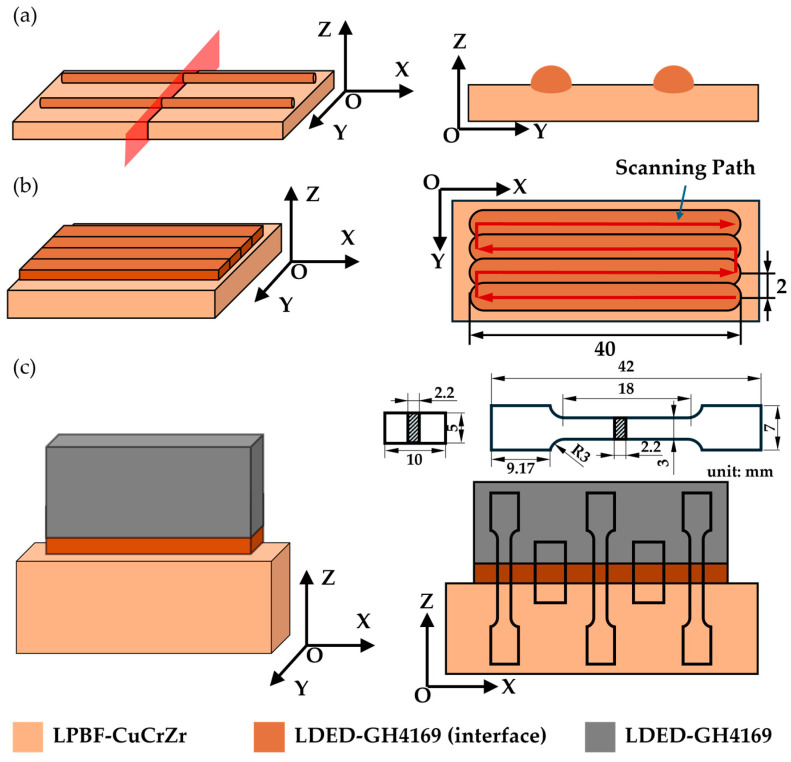
Schematic diagram of test specimens: (**a**) single-melt-track specimen and its cross-section; (**b**) single-layer specimen; (**c**) thin-walled specimen and its tensile test specimen.

Single-melt-track, single-layer, and thin-walled test specimens of CuCrZr/GH4169 were manufactured, as illustrated in Figure 3. For the melt track specimens, a series of process parameters were employed to deposit the GH4169 single melt track on the LPBF-manufactured CuCrZr matrix. Subsequently, the specimens were cut along a direction perpendicular to the melt track using wire cutting, allowing for the analysis of the single bead melt pool information through cross-sectional examination. In the case of the single-layer specimens, GH4169 was similarly deposited on the CuCrZr matrix using a range of process parameters, further exploring the process window. The thin-walled specimens were initially manufactured using the same interfacial process parameters as the single-layer specimens, depositing two layers on the CuCrZr matrix, followed by the deposition of GH4169 to complete the manufacturing of the thin-walled specimens. Wire electrical discharge machining was used to extract tensile test specimens and microstructural observation specimens (Figure 3c), ensuring that the interface was positioned at the center of both the tensile test specimens and the microstructure observation specimens.

### 2.3. Analysis and Testing Methods

The specimens were sequentially polished using 400-grit, 1000-grit, 2000-grit, and 4000-grit sandpaper, followed by polishing with a silica suspension of 0.5 μm and 0.05 μm. Subsequently, a mixed solution of hydrogen peroxide (3 vol.%) and hydrochloric acid (20 vol.%) in a ratio of H_2_O_2_:HCl = 3:2 was employed to etch both CuCrZr and GH4169, after which the specimens were cleaned with alcohol [19]. The interface metallography and microstructure were observed using a LEICA DMi8C optical microscope (OM) (Leica Microsystems GmbH, Wetzlar, Germany) and a Nova NanoSEM 430 Field Emission Scanning Electron Microscope (SEM) from (FEI Company, Hillsboro, OR, USA). The fracture morphology of the tensile specimens was examined with a Quanta200 environmental SEM (FEI Company, Hillsboro, OR, USA). Some specimens were subjected to argon ion polishing using a Hitachi IM4000plus ion milling system (Hitachi, Tokyo, Japan) under conditions of 4000 V and an angle of 3° for approximately 2 h. Subsequently, electron backscatter diffraction (EBSD) testing was conducted on the specimens using a GeminiSEM 300 field emission SEM (ZEISS, Oberkochen, Germany), equipped with an Oxford-SYMMETRY S2 EBSD detector (Oxford Instruments, Abingdon, UK), and the results were processed and analyzed using Channel5 software (Version 5.12.74.00).

The microhardness variation was measured using the MVS-1000D1 automatic turret digital microhardness tester (Shanghai Jimin Testing Instrument Co., Ltd., Shanghai, China) and the HMV-2T micro-Vickers hardness tester (SHIMADZU Corporation, Kyoto, Japan). Continuous measurements were conducted at 8 or 9 points near the interface along the building direction, with a spacing of 50 μm between adjacent points. Each specimen was tested in triplicate using a 0.2 kg load for 15 s, and the microhardness values were recorded at each indentation point. Tensile properties were evaluated using the CMT5105 electronic universal testing machine (Zhuhai Sansitai Electric Equipment Co., Ltd., Zhuhai, China) with testing conducted at room temperature.

## 3. Results

### 3.1. Single Melt Track of GH4169 Deposited on the CuCrZr Matrix

**Figure 4:**
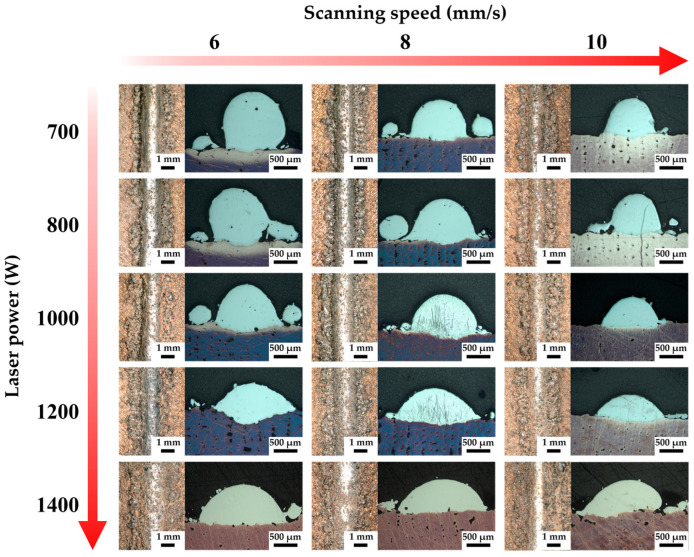
The influence of LDED process parameters on the bonding characteristics of the GH4169 single melt track deposited on CuCrZr matrix.

Figure 4 presents the optical micrographs of the single melt track of GH4169 deposited on a CuCrZr matrix under various LDED process parameters, illustrating the influence of these parameters on the bonding of the single melt track. The overall quality of the single melt track deposited by LDED was satisfactory, although a few process parameters resulted in minor porosity. In contrast, the CuCrZr substrate produced by LPBF was of lower quality, exhibiting a reduced density (more porosity). This is attributed to CuCrZr’s high reflectivity to infrared laser light, leading to insufficient energy input and a higher incidence of unmelted porosity defects during the manufacturing process. Within the range of process parameters selected for this experiment, the bonding condition at the CuCrZr/GH4169 interface was favorable, with virtually no porosity or crack defects observed. Under appropriate process parameter conditions, a defect-free bonding interface can be achieved.

**Figure 5:**
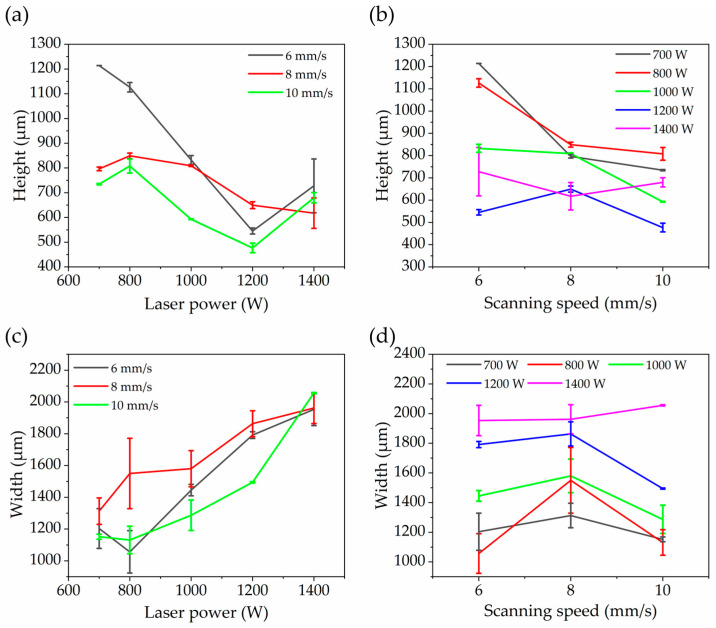
Effects of laser power and scanning speed on the height and width of a single melt track: (**a**) effect of laser power on the height of a single melt track; (**b**) effect of scanning speed on the height of a single melt track; (**c**) effect of laser power on the width of a single melt track; (**d**) effect of scanning speed on the width of a single melt track.

Figure 5a,b show the effect of laser power and scanning speed on the height and width of the single melt track of GH4169 formed on the CuCrZr substrate. The height of the single melt track was significantly affected by the process parameters. The highest height was about 1214 μm under the conditions of 700 W and 6 mm/s, while the lowest height was about 477 μm under the conditions of 1200 W and 10 mm/s. In general, the height of the single melt track decreased as the laser power increased, and it dropped significantly as the scanning speed increased; the latter was due to the reduced powder feed per unit distance at higher scan speeds. The width of the single melt track remained roughly between 1000 and 2000 μm and tended to increase with higher laser power. This is because higher laser power improves the melt pool’s wettability, causing the track width to increase accordingly. Scanning speed had little effect on the width of the single melt track.

### 3.2. Single-Layer Specimen of GH4169 Deposited on the CuCrZr Matrix

**Figure 6:**
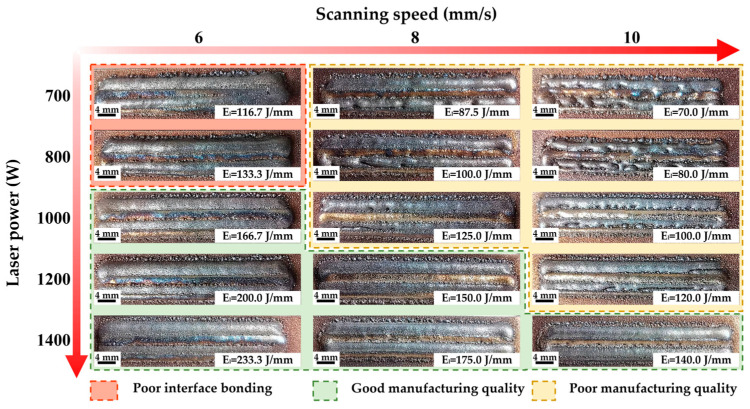
Surface of single-layer specimens of GH4169 deposited on the CuCrZr matrix under different LDED process parameters.

Figure 6 shows the surface conditions of single layer GH4169 specimens deposited on the CuCrZr matrix under different LDED processes. From the perspective of laser power, an increase in laser power can enable the melt pool to be fully wetted and spread, effectively improving the continuity of the melt track. Meanwhile, it can increase the width of a single melt track, thereby reducing the defects in the overlapping area between adjacent melt tracks and improving the flatness of the specimen surface. With regard to scanning speed, particularly at low laser power, increasing the speed significantly reduces the continuity of each melt track in the single-layer specimen. The melt track shows a tendency of sphericity, and the flatness of the melt track surface is reduced. Considering the combined effects of laser power and scanning speed, an analysis can be conducted based on the linear energy density (LED) of the laser, which is calculated using the following Equation (1) [20]:

(1) $$E_{l}=\frac{P}{v}$$

where P represents the laser power and v denotes the scanning speed. Due to the inherent high thermal conductivity and high reflectivity of the CuCrZr matrix manufactured by LPBF, insufficient energy input under low LED conditions results in inadequate melting and flow of the GH4169 powder. This leads to a tendency for sphericity in the melt pool, while the reduced melt pool width may decrease the overlap area between adjacent melt pools, potentially causing poor bonding between neighboring tracks and uneven surfaces in single-layer specimens. As illustrated in Figure 6, when the LED is less than 120 J/mm, particularly when it falls below 100 J/mm, the single-layer specimens, although fundamentally manufactured, exhibit subpar quality. However, under conditions of low laser power and low scanning speed, even though the LED may reach 133 J/mm, the insufficient laser power results in inadequate melting and bonding of the GH4169 and CuCrZr matrix. Concurrently, the significant accumulation of heat due to low scanning speed leads to a rapid increase in thermal stress, making it difficult to suppress edge warping under the combined effects of weak interfacial bonding and high thermal stress, thereby manifesting as poor bonding. Conversely, when the LED exceeds 140 J/mm, satisfactory bonding between the GH4169 and CuCrZr is achieved. Virtually no defects are observed in the overlap areas of adjacent melt tracks, thereby ensuring a flat specimen surface.

### 3.3. LLIAM of CuCrZr/GH4169 Thin-Walled Specimens

#### 3.3.1. Metallography

**Figure 7:**
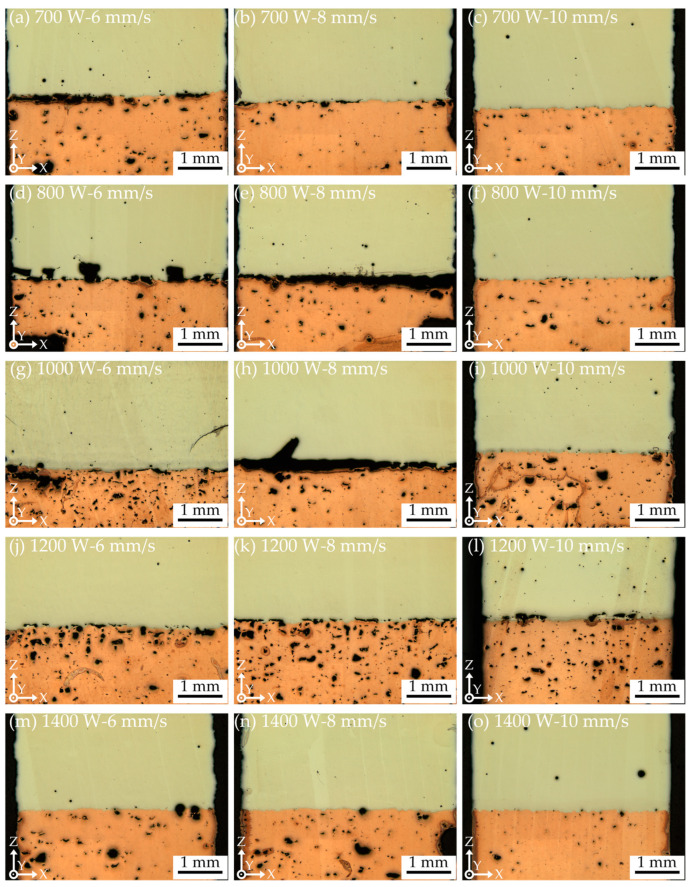
Metallography of the interface of CuCrZr/GH4169 specimens under different process parameters.

Figure 7 illustrates the interface of CuCrZr/GH4169 specimens under various processing parameters. Hole defects can be observed at the bonding interface across specimens subjected to different processing conditions; however, the dimensions of these holes are generally small, predominantly below 65 μm. Notably, under the conditions depicted in Figure 7g, a significantly higher number of holes are evident at the interface, with the largest holes reaching approximately 300 μm. Additionally, pronounced oblique cracks are observed in the GH4169 region. Figure 7d reveals the most severe hole defects, where, based on the presence of larger holes, there is also a critical lack of metallurgical bonding between the melt track and the matrix, both of which contribute to cracking at the interface of the specimen. Furthermore, in Figure 7b,g,j–l, although bonding between GH4169 and CuCrZr is achieved, a substantial number of interface cracks resulting from poor bonding are present, with lengths typically ranging from 200 to 500 μm. The aforementioned phenomena are exacerbated in Figure 7a,e,h, where cracks exceeding 2 mm in length have formed, some even penetrating through the entire specimen. At a laser power of 1400 W, the number of hole defects is relatively low, and no interface cracks are observed.

It is difficult to completely avoid interface hole defects in LLIAM-fabricated CuCrZr/GH4169 components; however, using a higher scanning speed can somewhat reduce the size and number of these defects. Significant defects primarily occur at laser powers below 1000 W, whereas higher laser powers promote good metallurgical bonding at the interface. This indicates that insufficient energy input at low laser power severely impairs the melting and bonding between CuCrZr and GH4169.

**Figure 8:**
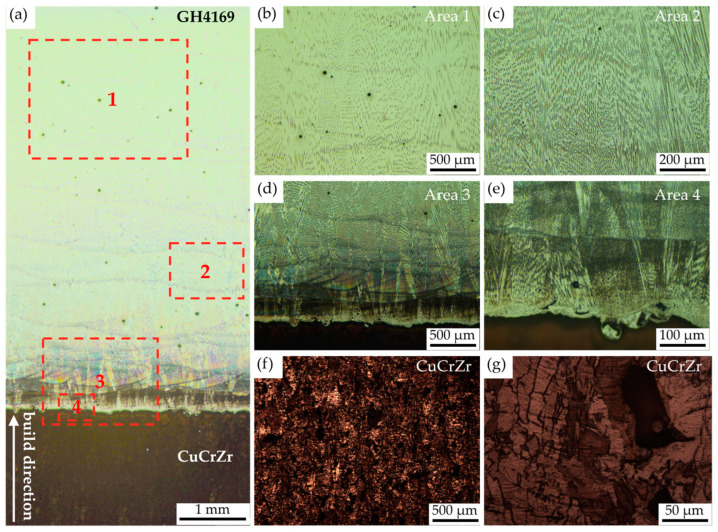
Typical metallographic images of a CuCrZr/GH4169 thin-walled specimen: (**a**) metallographic image of a thin-walled specimen; (**b**,**c**) metallographic image of the GH4169 region; (**d**,**e**) metallographic image of the interface; (**f**,**g**) metallographic image of the CuCrZr region.

Figure 8 shows a typical metallographic image of the interface of the CuCrZr/GH4169 thin-walled specimen. The LDED parameters at the interface are a laser power of 1400 W and a scanning speed of 10 mm/s. In the solid processing area of GH4169, no other defects were observed except for some micropores. This demonstrates that the chosen thin-walled processing parameters for GH4169 yield good build quality, and that this region has no significant effect on the component’s overall performance. Additionally, the dendrites in the GH4169 region grow predominantly along the building direction, and their size gradually increases with increasing deposition height. This can be attributed to the increase in deposition height accompanying the extended deposition time during the LDED of GH4169, which is also accompanied by a continuous input of energy. This process results in a gradual accumulation of heat, leading to a reduction in the temperature gradient of the melt pool and a more uniform temperature distribution, thereby allowing the dendrites more time for crystal growth during solidification [21].

In Figure 8a,d, it can be clearly observed that there are obvious differences between the interface region and the GH4169 region after corrosion. On the one hand, this is because the different thermal histories and thermal accumulations in the two regions result in relatively smaller dendrite sizes at the interface. On the other hand, it may be due to the change in the micro-composition of the interface region caused by the mixed transition between CuCrZr and GH4169 at the interface. Meanwhile, in Figure 8e, the melting and mixing of the two materials in the interface region can also be observed, such phenomena caused by the swirling flow of the molten metal in the melt pool due to the Marangoni convection during the deposition process.

In the CuCrZr region, the defects are distinctly evident. The inherent high reflectivity of the material results in limited absorption of laser energy by the CuCrZr powder during the manufacturing process. Additionally, its excellent thermal conductivity further contributes to the rapid dissipation of the absorbed energy in the form of heat. Consequently, the powder material struggles to achieve sufficient melting, leading to the formation of hole defects [22].

#### 3.3.2. Microstructure

**Figure 9:**
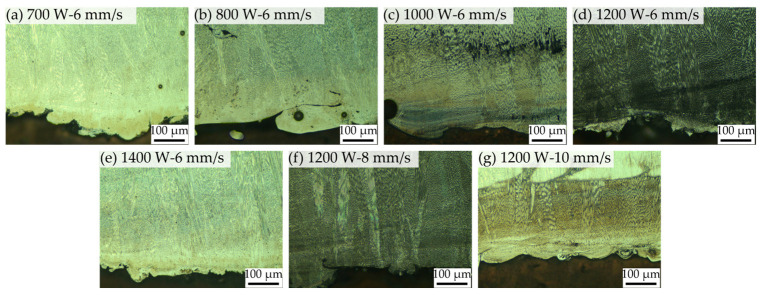
Microstructural characteristics of the interface in CuCrZr/GH4169 thin-walled specimens under different process parameters.

Figure 9 illustrates the microstructure of CuCrZr/GH4169 specimens under different processing parameters. The interfacial microstructures at varying laser power levels exhibit similarities, characterized by the presence of dendritic subgrains and cellular subgrains, with a noticeable trend of increasing subgrain size along the building direction, particularly evident in Figure 9c,d. This phenomenon can be attributed to the smaller temperature gradient at the upper region of the melt pool compared to its lower counterpart, which facilitates grain growth. Furthermore, as laser power increases, the interfacial microstructure near the CuCrZr side shifts from mostly cellular subgrains to predominantly dendritic subgrains. The enhancement of laser power effectively increases the thermal input during the deposition process, which reduces the temperature gradient in the melt pool region, thereby creating more favorable conditions for the growth of dendritic subgrains. For varying scanning speeds, the composition of the interfacial microstructure does not exhibit significant changes; however, a slight decrease in the density of subgrains and a marginal increase in their size can be observed. This alteration can likewise be attributed to the changes in the temperature gradient. Given that the interfacial region is deposited on a highly thermally conductive CuCrZr matrix, heat can be rapidly transferred into the bulk material at high scanning speeds, resulting in a relatively uniform temperature distribution in the melt pool region and consequently lower temperature gradients. Therefore, with the increase in scanning speed, although the overall temperature may be relatively lower, the temperature gradient in the melt pool slightly decreases, which is more conducive to grain growth and results in relatively larger subgrain sizes.

**Figure 10:**
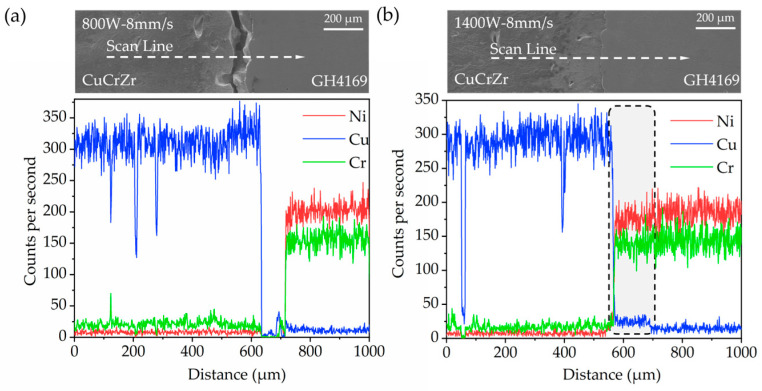
EDS line scan of the interface of CuCrZr/GH4169 thin-walled specimens under different process parameters: (**a**) 800 W, 8 mm/s; (**b**) 1400 W, 8 mm/s.

An energy dispersive spectrometer (EDS) line scan was conducted on the interfaces of CuCrZr/GH4169 thin-walled specimens under certain interface process parameters to observe the elemental variations at the multi-material interfaces, as illustrated in Figure 10. It can be observed that under the low laser power processing conditions represented by 800 W and 8 mm/s, there is poor bonding between the CuCrZr and GH4169 materials, resulting in the presence of a region in Figure 10a where no elements were detected. The elemental contents of Ni, Cu, and Cr on either side of the crack exhibit negligible variations, indicating a sharp change in elemental composition at the bonding interface. There is an absence of a transitional phenomenon of elemental variation in the interface region, which suggests that the LDED deposition process of GH4169 under these conditions shows significant deficiencies in the molten mixing with CuCrZr. Consequently, there is almost no transitional mixing between the two materials, leading to inadequate bonding at their interface. In contrast, under the high laser power processing conditions represented by 1400 W and 8 mm/s, the elements Ni and Cu at the interface region also exhibit a sharp change; however, a small segment of approximately 100 μm in length shows a stepped curve. This indicates that under these processing parameters, a transitional mixing region of elements has emerged at the bonding interface, resulting in good bonding at the interface.

**Figure 11:**
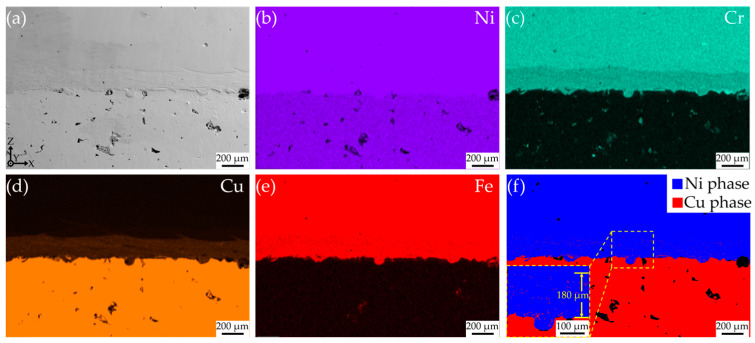
EDS surface scan and phase diagram of CuCrZr/GH4169 thin-walled specimens: (**a**) Interface SEM image; (**b**) Interface Ni element distribution; (**c**) Interface Cr element distribution; (**d**) Interface Cu element distribution; (**e**) Interface Fe element distribution; (**f**) Interface phase diagram.

Figure 11 presents the elemental EDS surface scan of the interfacial region under processing conditions of 1200 W and 10 mm/s. A distinct transitional zone for the elements Cr, Cu, and Fe can be observed at the interface. In the phase distribution map shown in Figure 11f, a striped region indicative of the Cu phase is detected within the Ni phase near the interface, with a width of approximately 180 μm. The location and width of this region correspond to the elemental transitional zone depicted in the EDS surface scan. Furthermore, the morphology of the Cu phase supports the existence of Marangoni convection and the segregation of elements within the interfacial region.

**Figure 12:**
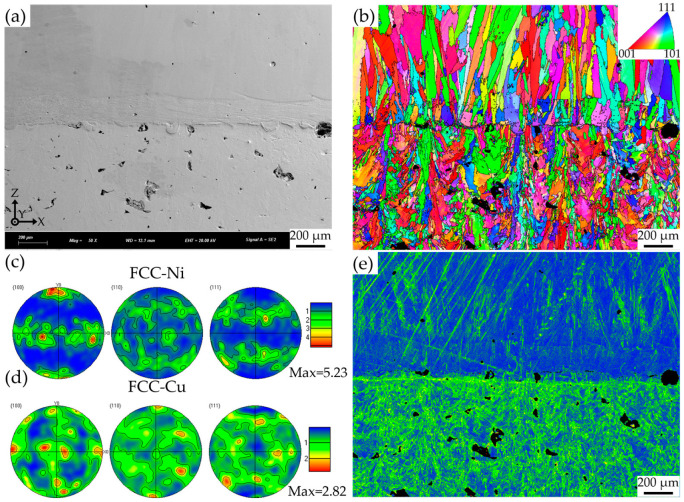
Grain orientation distribution, microstress, and texture strength at the interface of CuCrZr/GH4169 thin-walled specimens: (**a**) interface SEM image; (**b**) interface inverse pole figure; (**c**) GH4169 pole figure; (**d**) CuCrZr pole figure; (**e**) interface kernel average misorientation.

Figure 12 illustrates the grain orientation distribution, microstress, and texture strength in the interface region of the CuCrZr/GH4169 thin-walled specimen manufactured using a 1200 W, 10 mm/s interface process. This serves as a representative analysis of the LLIAM specimens in this study. In the GH4169 region, the grain orientation predominantly exhibits a strong <001> orientation alongside a somewhat weaker <101> orientation, with a maximum pole density reaching 5.23. Notably, this preferred orientation does not display a tendency to change with increasing deposition height. Conversely, the CuCrZr region does not exhibit a very strong preferred orientation; although the pole densities for the <001> and <101> orientations are relatively high, the maximum pole density is only 2.82. Generally, the emergence of a strong <001> preferred orientation in additive manufacturing specimens is a common occurrence, influenced by the maximum thermal flow direction (typically aligned with the building direction) during the laser additive manufacturing process. During laser scanning, the thermal intensity is highest at the current melt pool, while previously scanned regions cool. This thermal gradient may slightly deflect the melt pool’s heat flow, causing some grains to develop a <101> preferred orientation. Regarding the microstress conditions of the specimen, the Kernel Average Misorientation (KAM) map shown in Figure 12d clearly indicates that the density of the blue region in the CuCrZr area is lower, suggesting a relatively high geometric dislocation density and consequently higher microstress, particularly near the unmelted holes. The GH4169 region exhibits significantly lower microstress levels, especially in the interface transition zone. However, elevated microstress is observed on the upper side of the transition zone, along with similarly high stress in the region directly adjacent to the CuCrZr. This phenomenon is attributed to higher microstress in the direct contact area, caused by differences in element distribution and material properties between the two adjacent regions. In contrast, the interior of the interface transition zone has a more uniform elemental distribution, resulting in lower stress in that region.

**Figure 13:**
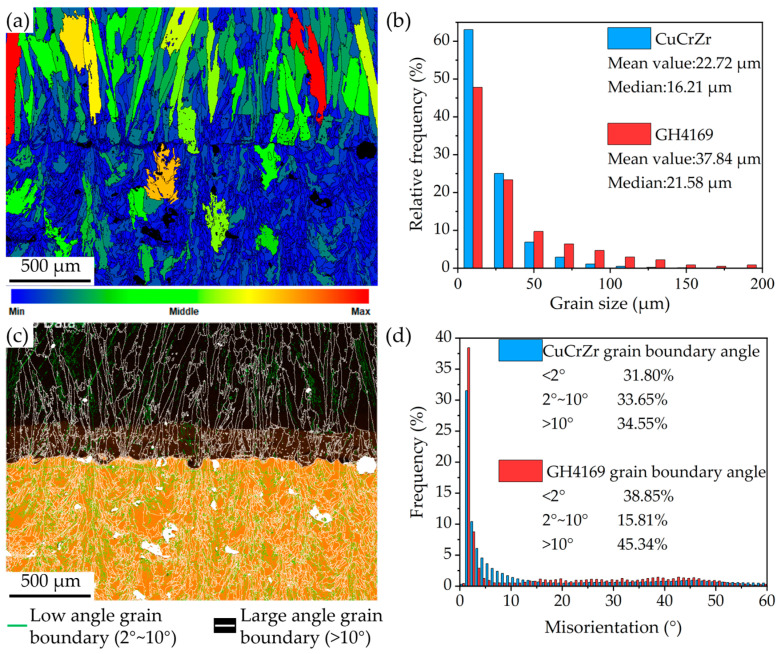
Statistics of grain size and grain boundary angle at the interface of CuCrZr/GH4169 thin-walled specimens: (**a**) grain size distribution diagram; (**b**) grain size statistical chart; (**c**) grain boundary angle distribution diagram; (**d**) grain boundary angle statistical chart.

Figure 13 shows the interface grain size and grain boundary angle of the specimen. The grains in the CuCrZr region are mainly small equiaxed grains, with an average value of 22.7 μm and a median of 16.2 μm. The grains in the GH4169 region are mainly large columnar grains, with an average value of 37.8 μm and a median of 21.6 μm. This difference in grain size is mainly caused by the different AM methods adopted for the two materials. The smaller melt pool and larger cooling rate during the LPBF of CuCrZr result in small equiaxed grains. The LDED, due to the larger laser spot diameter, higher power, and relatively smaller temperature gradient, tends to generate large columnar grains along the building direction. However, compared to GH4169 at higher deposition heights, the grains in the CuCrZr/GH4169 interface region are smaller and more equiaxed. This is mainly because the excellent thermal properties of the CuCrZr matrix lead to a larger temperature gradient compared with the subsequent LDED deposition process. In the CuCrZr region, low-angle grain boundaries dominate, indicating small orientation differences between grains. This typically leads to weak anisotropy and a low texture intensity. In the GH4169 region, the subgrain boundaries with an orientation difference of <2° account for the largest proportion, approximately 38.85%, indicating that there are a large number of subgrains with relatively consistent grain orientations in this region, which is beneficial for the formation of large-sized columnar grains. Meanwhile, the GH4169 region contains a higher fraction of high-angle grain boundaries, indicating larger orientation differences between its large grains and more pronounced anisotropy. This is consistent with the texture observed in the CuCrZr/GH4169 specimen (Figure 12c,d).

#### 3.3.3. Mechanical Properties

**Figure 14:**
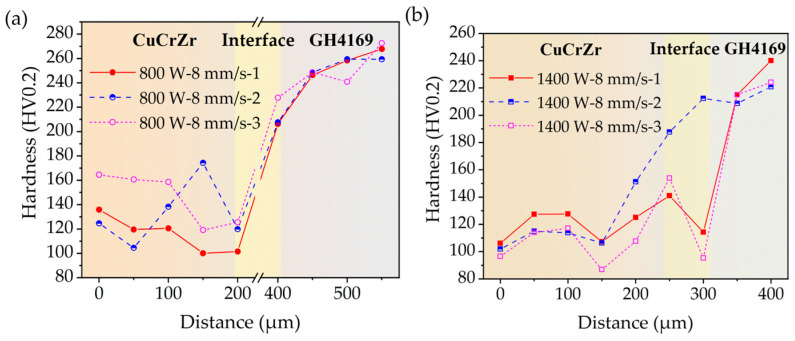
Microhardness at the interface of CuCrZr/GH4169 thin-walled specimens: (**a**) 800 W, 8 mm/s; (**b**) 1400 W, 8 mm/s.

Figure 14 illustrates the microhardness variation curve of the interface region of representative CuCrZr/GH4169 thin-walled specimens. For the stable manufacturing regions of CuCrZr and GH4169 in this study, the measured average hardness of the two materials is approximately 120–140 HV and 230–260 HV, respectively. In related studies, the hardness of CuCrZr is generally reported to be in the range of 120–150 HV, while that of GH4169 is between 260–320 HV, indicating that the hardness of the materials manufactured in this study is comparable to the findings of other researchers [2,23,24]. Regarding the hardness variation at the interface, it is evident that the changes in the microhardness curve are consistent with the elemental composition variations shown in Figure 10. In the specimen with process parameters of 800 W and 8 mm/s, the hardness on the CuCrZr side of the interface region exhibits a slight decline, while the hardness on the GH4169 side experiences a sudden increase, which further escalates slightly with the rise in deposition height. The reduction in hardness on the CuCrZr side is likely attributed to the thermal effects of the LDED process. The abrupt change in hardness at the interface further corroborates the notion that the transition zone between the dissimilar materials under these parameters is quite limited. For the specimen with process parameters of 1400 W and 8 mm/s, a noticeable trend of gradually increasing microhardness is observed in the vicinity of the interface, indicating a favorable gradient transition in performance within the interface region under high laser power. This suggests a good compositional mixing and metallurgical bonding between CuCrZr and GH4169.

**Figure 15:**
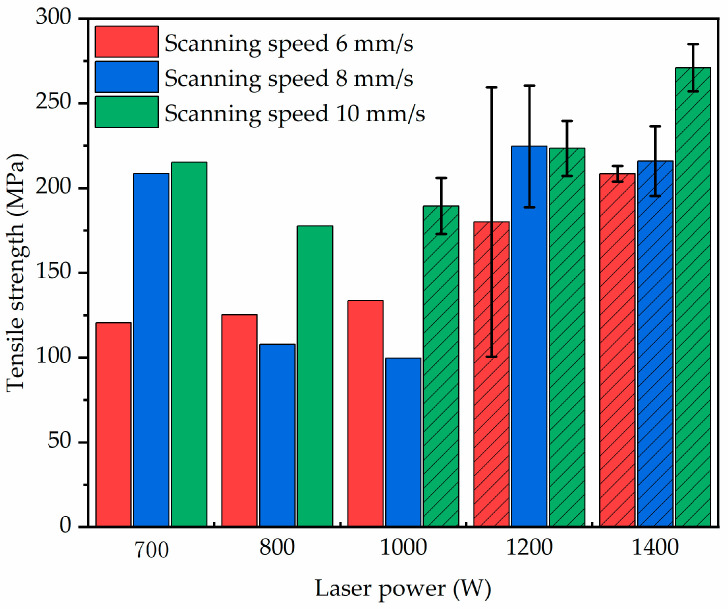
The fracture characteristics and tensile strength of CuCrZr/GH4169 tensile specimens (with slashes indicating that a fracture occurred in CuCrZr).

Figure 15 presents the tensile fracture characteristics and tensile strength of CuCrZr/GH4169 specimens manufactured using different interface processes in this study. It can be observed that under laser power conditions below 1000 W, the CuCrZr/GH4169 specimens typically exhibit fractures at the interface, with some specimens at scanning speeds of 6 mm/s and 8 mm/s demonstrating tensile strengths even lower than 150 MPa, indicating poor interface bonding effectiveness. In contrast, at laser power levels of 1200 W and 1400 W, the tensile strength of the specimens shows an improvement compared to the low power conditions, with fracture primarily occurring on the CuCrZr side. This suggests that the bonding strength of the interface under these processing conditions exceeds the strength of CuCrZr. On one hand, specimens with numerous defects under different interface processes tend to fracture at the interface. On the other hand, as discussed in Section 3.3.2, lower laser power is more conducive to the formation of cellular subgrains, which, while promoting plasticity through the generation of numerous subgrain boundaries, can also lead to fracture. Conversely, higher laser power tends to favor the formation of dendritic subgrains, which contribute to toughening and elongation during the tensile process, thereby helping to delay material fracture. Furthermore, for the processing parameters that yield good interface bonding, the tensile tests in this study typically resulted in fractures occurring on the CuCrZr side, with an average tensile strength of approximately 234 MPa, which can be regarded as the tensile strength of the CuCrZr matrix. Compared with existing studies [25,26,27,28], this strength is within the normal range of the ultimate tensile strength of LPBF-formed CuCrZr.

**Figure 16:**
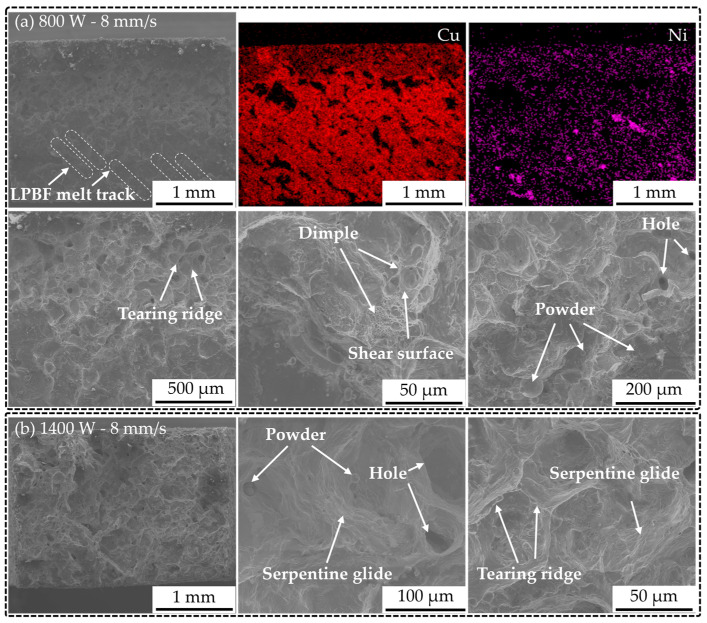
CuCrZr/GH4169 tensile fracture morphology: (**a**) 800 W, 8 mm/s; (**b**) 1400 W, 8 mm/s.

To analyze the fracture mechanisms of the CuCrZr/GH4169 specimens in depth, we observed tensile fracture surfaces at different fracture locations. Figure 16 illustrates two representative fracture surfaces. For the tensile fracture of the specimen with deposition process parameters of 800 W and 8 mm/s, the EDS elemental mapping results revealed the presence of both Cu and Ni elements, alongside the observation of LPBF melt tracks at the fracture site, indicating that the fracture location corresponds to the CuCrZr/GH4169 interface. Further examination of the SEM images of the tensile fracture at the interface revealed pronounced tearing ridges and shear facets, which are characteristic features of quasi-cleavage fracture. Additionally, a small number of fine ductile dimples were observed, suggesting that the tensile fracture of this specimen is a result of the combined effects of ductile and brittle fracture, with a predominant manifestation of brittle fracture. In the case of the tensile fracture occurring within the CuCrZr region (Figure 16b), similar quasi-cleavage fracture features were noted, but a significant presence of serpentine glide was also observed, indicative of ductile fracture. Therefore, it can be concluded that the tensile fracture of this specimen is primarily ductile in nature, attributed to the high elongation capacity of CuCrZr.

## 4. Discussion

### 4.1. Compatibility of CuCrZr/GH4169 Multi-Material Structures

**Figure 17:**
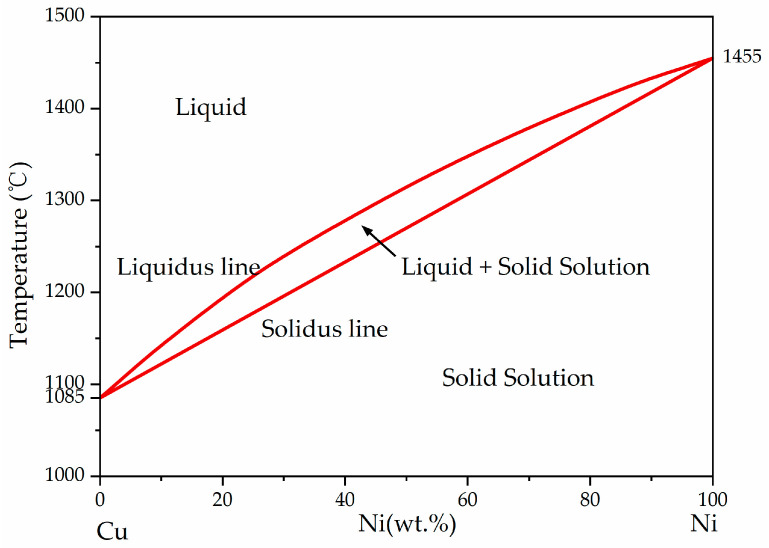
Cu-Ni binary phase diagram [29,30].

Within the relatively wide range of process parameters adopted in this study, good bonding can be achieved between CuCrZr and GH4169. This is due to the similar atomic radii of Cu (atomic number 29) and Ni (atomic number 28), the same face-centered cubic (FCC) crystal structure, similar chemical properties including electron cloud distribution and chemical bond properties, and the relatively small heat of dissolution between them. As a result, the two elements can occupy similar positions in the crystal structure without causing significant stress or distortion, and they are more likely to diffuse and mix with each other in the solid state without requiring a large amount of energy. Figure 17 shows the Cu-Ni binary phase diagram. Theoretically, Cu and Ni can be infinitely miscible at high temperatures, and there is only a single-phase alloy between them. In Figure 11, the Cu-rich area is uniformly and abundantly distributed with Ni element, while other main elements in GH4169 such as Cr and Fe are hardly detected in this area. Therefore, benefiting from the excellent elemental compatibility of Cu and Ni, CuCrZr and GH4169 exhibit good compatibility [29,30].

### 4.2. Behavior of the Melt Pool and Defect Formation in a Matrix with High Thermal Conductivity and High Reflectivity

In this study, the formation of hole defects at the CuCrZr/GH4169 interface was almost unavoidable—these holes are among the common defects in LDED processes. This phenomenon is primarily attributed to two factors: Firstly, during the melting process, selective evaporation of elements from the alloy or entrapment of protective gas within the melt pool occurs. Secondly, insufficient energy input leads to unmelted voids [31].

**Figure 18:**
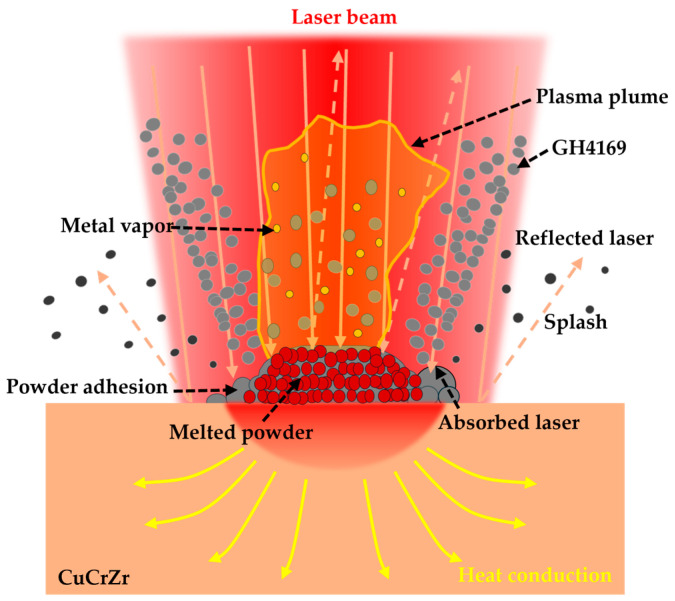
Effect of the high-thermal-conductivity and high-reflectivity CuCrZr matrix on the deposition of GH4169.

Figure 18 illustrates the impact of the high thermal conductivity and high reflectivity matrix CuCrZr on the deposition of GH4169. Due to the inherent high reflectivity of the CuCrZr matrix manufactured by LPBF, a significant amount of the laser beam is reflected when it strikes the CuCrZr surface, resulting in a corresponding inability of the laser energy to be absorbed by the powder and the melt pool. Furthermore, since the energy distribution of the laser beam follows a Gaussian profile, the energy density is higher in the central region while it is lower in the peripheral areas. Consequently, the powder located in the outer regions of the laser beam is capable of absorbing significantly less energy for melting purposes. Simultaneously, due to the exceptional thermal properties of CuCrZr, the energy absorbed by the melt pool is rapidly conducted to deeper and broader areas within the CuCrZr matrix. This implies that the laser melt pool has a larger heat-affected zone; however, this also results in a reduction of the absorbed laser energy available for melting both the powder and the solidified material, thereby hindering the accumulation of heat within the melt pool to reach the melting point of the powder and the already solidified material. Under the combined effects of the aforementioned factors, only the central region with higher laser energy is capable of fully melting the GH4169 powder, while the regions adjacent to the melt pool, due to insufficient energy input, fail to adequately melt the powder, leading to severe powder adhesion and unmelted particles, as well as a narrower melt track. Additionally, the reflected energy induces thermal effects, complicating and rendering the temperature and flow fields within the laser heating region more heterogeneous, which can easily result in thermal damage, deformation, or thermal cracking [32]. For example, in Figure 7g,n, it can be observed that crack defects appear inside the GH4169 located above the multi-material interface. This is because when GH4169 is formed near the interface, the high thermal conductivity of the CuCrZr matrix has a greater influence on the temperature gradient of the molten pool. In addition, the difference in thermal expansion coefficients between CuCrZr and GH4169 leads to large local thermal stresses during the cooling of the molten pool in this area, in turn leading to the appearance of cracks. This is also confirmed by the KAM diagram in Figure 12e.

The ratio ΔH/h_s_, as shown in Equation (2), can be used to determine the heat transfer mode; a larger ratio favors the occurrence of the keyhole mode, while a smaller ratio tends to maintain the conduction mode [33,34].

(2) $$\begin{array}{c} \frac{\Delta H}{h_{s}}=\frac{\mathrm{AP}}{\pi h_{s}\sqrt{\mathrm{Dv}\sigma^{3}}} \end{array}$$

Here, A represents the laser absorptivity, P is the laser power, D is the material’s thermal diffusivity, v is the laser scanning speed, and σ denotes the laser spot size. Under conditions of constant laser power, scanning speed, and laser spot size, the higher thermal conductivity of the CuCrZr matrix and its lower absorptivity to infrared lasers lead to a smaller ΔH/h_s_ ratio. This results in the melt pool’s heat transfer mode favoring conduction. In other words, the high thermal conductivity of CuCrZr allows only a limited amount of energy to melt the powder and solid material. Although a larger heat-affected zone is achieved, the insufficient heat accumulation in the melt pool prevents the CuCrZr matrix from melting over a broader area. Consequently, during single-track formation with selected process parameters, only a small portion of the CuCrZr matrix surface melts and mixes with the molten GH4169 in the melt pool, resulting in a shallow melt pool with low dilution. For instance, Figure 7h displays a pronounced and severe arcuate defect, with a width of up to 500 μm. The shape of this defect closely resembles that of the melt pool’s sidewalls. This observation suggests that inadequate energy absorption by the melt pool during the deposition process leads to poor bonding between the melt pool and the matrix. Combined with cooling shrinkage, thermal stress, and the high thermal conductivity of CuCrZr, this results in poor interfacial bonding.

### 4.3. Stress at the CuCrZr/GH4169 Multi-Material Interface

Related studies have explained the relationship between interfacial stress and factors such as thermal expansion coefficient through the temperature gradient mechanism, as shown in Equation (3) [35]:

(3) $$\begin{array}{c} \sigma =\frac{\Delta T}{E}\left(\alpha_{2}-\alpha_{1}\right) \end{array}$$

where σ is the stress, ΔT is the temperature change, α_1_ and α_2_ are the thermal expansion coefficients of the two materials, and E is the average elastic modulus of the materials. This indicates that differences in thermal expansion coefficients between the two materials and temperature gradient differences caused by varying thermal conductivities can lead to stress concentration at the interface, resulting in severe cracking or delamination at the interface, which is a significant factor contributing to the performance degradation of dissimilar material additive manufacturing components [36,37]. Under low laser power processing conditions, the temperature variation at the CuCrZr/GH4169 interface is greater, likely due to insufficient energy input and the high thermal conductivity of the matrix, leading to a lower temperature in CuCrZr. The large temperature difference at the interface between the two materials causes a higher temperature gradient. In other words, under otherwise identical conditions, a larger temperature gradient under low laser power processing leads to greater interfacial stress, making the bonding interface more prone to poor bonding and cracking. This is clearly shown in Figure 7.

### 4.4. Thermal Conductivity of CuCrZr/GH4169 Multi-Materials

The thermal conductivities of GH4169 and CuCrZr, denoted as λ_g_ and λ_c_, respectively, can be obtained using Equations (4) and (5). The thermal conductivity, λ_b_, of the CuCrZr/GH4169 dissimilar material interface can be determined by solving Equations (4)–(7) simultaneously [2,38,39]:

(4) $$\begin{array}{c} \lambda_{g}\left(T\right)=5.291+0.0152T+1.382\times 10^{-6}T^{2},\;298\leq T\leq 800\;K \end{array}$$

(5) $$\begin{array}{c} \lambda_{c}\left(T\right)=354.414+5.744\times 10^{-2}T\;-\;2.928\times 10^{-4}T^{2}+3.532\times 10^{-7}T^{3},\;T\leq 500\;{}^{\circ}C \end{array}$$

(6) $$\begin{array}{c} R=\frac{\Delta x}{\lambda A} \end{array}$$

(7) $$\begin{array}{cc} R_{b}=R_{c}+R_{g} & \Rightarrow \;\frac{\Delta x_{b}}{k_{b}A}=\frac{\Delta x_{c}}{\lambda_{c}A}+\frac{\Delta x_{g}}{\lambda_{g}A}\\ \; & \Rightarrow \;\frac{1}{\lambda_{b}}=\left[\frac{\Delta x_{c^{\prime }}}{\lambda_{c}}+\frac{\Delta x_{g^{\prime }}}{\lambda_{g}}\right]\\ \; & \Rightarrow \;\lambda_{b}=\frac{\lambda_{c}\;\times \;\lambda_{g}}{\Delta x_{c^{\prime }}\;\times \;\lambda_{g}+\Delta x_{g^{\prime }}\;\times \;\lambda_{c}} \end{array}$$

where Δx_c’_ = Δx_c_/Δx_b_, Δx_g’_ = Δx_g_/Δx_b_, and λ represents the material’s thermal conductivity, A is the cross-sectional area, R denotes thermal resistance, and Δx denotes the material’s thickness.

**Figure 19:**
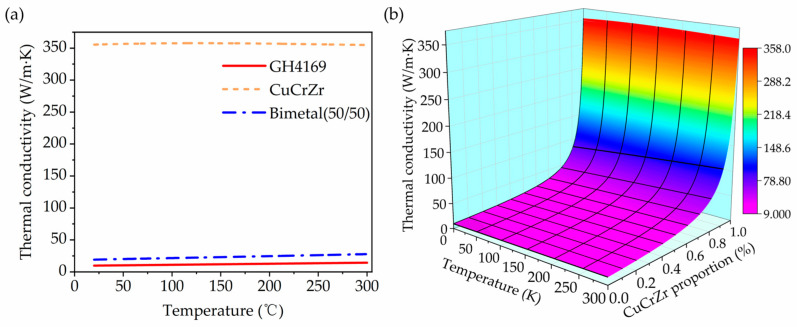
Thermal conductivity of CuCrZr, GH4169, and CuCrZr/GH4169 multi-materials: (**a**) thermal conductivity of GH4169, CuCrZr, and GH4169/CuCrZr (50/50) bimaterial; (**b**) thermal conductivity of GH4169/CuCrZr bimaterial at different temperatures and CuCrZr proportions.

The thermal conductivity calculation results of CuCrZr, GH4169, and their bimaterial system at different temperatures and thickness ratios are shown in Figure 19. Clearly, the thermal conductivity of CuCrZr remains around 354 W/m·K in the temperature range of 20 °C to 300 °C, significantly higher than that of GH4169. For the CuCrZr/GH4169 bimaterial with a 50/50 thickness ratio, the thermal conductivity can reach approximately twice that of GH4169, thereby substantially enhancing the thermal performance of the component.

The calculation results also indicate that the thickness ratio of the two materials significantly affects the thermal conductivity of the bimaterial component. As the proportion of CuCrZr increases, the thermal conductivity rises, especially when its thickness ratio exceeds 0.8, leading to a substantial improvement in thermal conductivity. However, this enhancement may be accompanied by a decrease in strength. Therefore, during the design process, it is essential to balance mechanical and thermal performances to ensure that the bimaterial component meets the requirements of its intended application.

## 5. Conclusions

This study investigates the integration of LPBF and LDED additive manufacturing processes for CuCrZr/GH4169 bimaterial components, focusing on their forming quality and performance control methods. The main conclusions are as follows:

1. Due to the good compatibility between Cu and Ni at high temperatures, GH4169 and CuCrZr can bond effectively over a wide range of process parameters. At laser line energy densities above 140 J/mm, a strong bond is achieved with minimal defects in the overlap regions between adjacent melt tracks. However, at low laser powers, issues such as porosity, poor bonding, and even cracking occur. In these cases, the interface lacks a distinct transition zone, and the hardness variation across the interface is abrupt. Conversely, high laser power results in a better interface, with Cu segregation phases present in the transition zone, measuring 100–200 μm in width. The microhardness profile near the interface shows a gradual increase, and tensile tests typically fracture on the CuCrZr side, with an average tensile strength of 234 MPa.
2. Mechanism of Defect Formation at the Interface Clarified for High-Conductivity Substrate. Systematic analysis revealed that CuCrZr’s high thermal conductivity (354 W/m·K) and low infrared absorptivity limited melt pool energy accumulation, leading to incomplete fusion and crack formation under low laser power. Elevated laser power (≥1200 W) suppressed such defects by shifting the melt pool behavior toward sufficient mixing and Marangoni-driven convection, enabling stable LDED on reflective substrates.
3. Thermal Conductivity and Mechanical Performance Balance: Adjusting the thickness ratio of GH4169 and CuCrZr in the bimaterial structure effectively tunes the thermal conductivity. A 50/50 thickness ratio achieves a thermal conductivity approximately twice that of GH4169. However, enhancements in thermal conductivity are often accompanied by reductions in mechanical strength. Therefore, balancing thermal and mechanical properties is crucial to meet the practical requirements of bimaterial components.

Importantly, the CuCrZr/GH4169 multi-material structure shows great promise for aerospace applications that demand efficient heat dissipation and mechanical durability. This study contributes to the state of the art by providing a viable manufacturing route for such functionally integrated structures, paving the way for lighter, more efficient thermal management solutions in next-generation aero-engines and space propulsion systems.

Despite the progress described above, this study still has several limitations that warrant further investigation in future work. First, the long-term performance of the heterogeneous material interface was not assessed: this study focused primarily on interface formation quality and static mechanical properties, but did not address the long-term stability of the CuCrZr/GH4169 interface under service conditions such as high temperature and cyclic loading. Future work should include experimental studies on the interface’s high-temperature endurance, thermal fatigue resistance, and oxidation behavior to evaluate its reliable service life under practical operating conditions. Second, regarding process optimization and consistency, the LLIAM process remains complex and requires further improvement in terms of stability and repeatability. Subsequent research could incorporate process monitoring and numerical simulation techniques to accurately predict and control melt pool dynamics and stress fields, thereby optimizing the process window and validating its consistency in larger-scale and more complex components.

## Figures and Tables

**Table 1:** Composition of CuCrZr and GH4169 (wt.%).

CuCrZr (LPBF)	Cr	Zr	Fe	Si	P	Cu
	0.5–1.5	0.05–0.25	<0.05	<0.05	<0.01	Bal
GH4169 (LDED)	C	Cr	Ni	Nb	Mo	Ti
	0.02–0.06	17.0–20.0	50.0–55.0	5.0–5.5	2.8–3.3	0.65–1.15
	Al	Co	Si	Mg	Cu	Fe
	0.2–0.8	≤1.00	≤0.35	≤0.01	≤0.3	Bal

**Table 2:** Process parameters of CuCrZr/GH4169 manufactured by LLIAM.

Process Parameters	CuCrZr (LPBF)	GH4169 (LDED)	
		Interface Integration Process	Thin-Walled Solid Process
Laser power (W)	400	700, 800, 1000, 1200, 1400	1000
Scanning speed (mm/s)	900	6, 8, 10	10
Scanning spacing (mm)	0.11	2	—
Layer height (mm)	0.03	—	0.36
Powder feed rate (g/min)	—	11.76	
Scanning strategy	Partition and interlayer orthogonal scanning	Interlayer bidirectional scanning	

## Data Availability

The original contributions presented in this study are included in the article. Further inquiries can be directed to the corresponding authors.
